# The changing landscape of Fabry disease: Impact of the inclusion of the GLA-gene in broader NGS or WES based panels on the phenotypic spectrum

**DOI:** 10.1371/journal.pone.0358572

**Published:** 2026-09-16

**Authors:** Laura van Dussen, Emilie V. Albers, Saskia N. van der Crabben, Ronald H. Lekanne Deprez, Astrid S. Plomp, Mirjam Langeveld

**Affiliations:** 1 Department of Endocrinology and Metabolism, Amsterdam UMC, Amsterdam Gastroenterology Endocrinology Metabolism (AGEM) Research Institute, University of Amsterdam, Amsterdam, The Netherlands; 2 Department of Clinical Genetics, Maastricht University Medical Center, Maastricht, The Netherlands; 3 Department of Clinical Genetics, Amsterdam UMC, University of Amsterdam, Amsterdam, The Netherlands; Universidade do Algarve, PORTUGAL

## Abstract

Fabry disease (FD) is the most common lysosomal storage disorder in which a severe, classical phenotype as well as a milder, non-classical phenotype can be distinguished. In this study we investigated the impact of the introduction of broader DNA sequencing techniques and subsequently the addition of the GLA-gene to NGS or WES based panels on the type of FD patient that is diagnosed by analyzing changes in the composition of the Dutch Fabry cohort over time. The current study confirms that Fabry disease is a genetically heterogeneous disorder with 64 different *GLA* variants established in a cohort of 319 patients. The introduction of broader DNA sequencing techniques, applied to a broader range of individuals with less specific symptoms, results in the identification of a higher proportion of individuals with less deleterious GLA variants and consequently a milder clinical phenotype. For the majority of individuals identified using the broader sequencing techniques cardiomyopathy is the presenting and only symptom of the disorder, in contrast to the multisystem classical Fabry disease phenotype. This phenotypic shift should be taken into account when comparing current to historical clinical and treatment effect data and requires tailored genetic counseling and clinical follow-up to prevent both over- and undertreatment.

## Introduction

Fabry disease (FD, OMIM #301500) is the most common (X-linked) inherited lysosomal storage disorder and results from a deficiency in the enzyme alpha-galactosidase A, caused by deleterious variants in the *alpha-galactosidase (GLA)-gene* (OMIM #300644). FD is a genetically heterogeneous disorder with over 1000 unique disease causing variants in the GLA-gene described to date [1]. The estimated prevalence of FD varies, which is largely the result of differences in disease definition and phenotypic heterogeneity of the disorder.

The classical FD phenotypes observed in male patients with very low, or absent enzyme activity, can be recognized by a triad of childhood onset symptoms: *neuropathic pain* in the extremities *(acroparesthesia)* which may be provoked by fever or physical exertion, *cornea verticillata* in the eyes and *angiokeratoma* in the skin. Complications of the disorder, which become clinically apparent from the fourth or fifth decade onwards, include renal failure, hypertrophic cardiomyopathy, cardiac rhythm disturbances, heart failure, myocardial infarction and stroke. Biochemically, substrate accumulation can be demonstrated by measuring high levels of globotriaosylceramide (Gb3) and globotriaosylsphingosine (lyso-Gb3, generally above 40 mmol/L) [2].

In addition to this well-defined, well understood and relatively homogeneous form of the disease, there are individuals with significant (albeit variable) residual enzyme activity levels, lower Gb3 and lyso-Gb3 substrate levels and therefore a milder phenotype. This group includes male patients with less deleterious GLA variants, as well as female patients with deleterious GLA variants causing classical disease in males (milder in women because of the compensatory unaltered GLA copy on the second X chromosome). The disorder in this group of patients is often described as non-classical, atypical, or cardiac variant FD. In the female patients with severe GLA variants, cornea verticillata or acroparesthesia can be present, but angiokeratoma are seldom observed and the clinical course is dominated by cardiac manifestations. In both groups (females with deleterious variants and male patients with less deleterious variants) cardiac complications occur approximately a decade later compared to male patients with classical disease [3], while renal and cerebral complications are far less prevalent. Clinical symptoms are hardly observed in female patients with less deleterious variants (leading to non-classical disease in males)or occur at an age at which complications may also be observed in a healthy cohort [3].

Clinical suspicion of FD, should be confirmed genetically and this also serves to facilitate cascade genetic screening. Possibilities of broad DNA sequencing techniques in diagnostics have evolved over the past decade. While targeted testing of the GLA-gene by Sanger sequencing has been possible since the early 1990s, broader DNA sequencing techniques including Next Generation Sequencing (NGS) and Whole Exome Sequencing (WES) have been offered as diagnostic tools in Amsterdam UMC from approximately 2011 onwards. These techniques enable sequencing of a large number of genes, or even an (almost whole) exome or genome, in a single analysis. Analysis can be restricted to genes associated with specific signs or symptoms (panel analysis, e.g., for hypertrophic cardiomyopathy), can include all OMIM-associated disease genes (mendeliome analysis), or (mostly in research settings) identify candidate genes (open exome/genome analysis). Following the introduction of these techniques, the GLA-gene has been added to several NGS or WES based panels such as (hypertrophic) cardiomyopathy, renal disorders and polyneuropathies.

The objective of this article is to describe the genetic and phenotypic heterogeneity in a large, single country FD cohort and to study the impact of the introduction of broader DNA sequencing techniques and subsequent inclusion of the GLA-gene in several NGS or WES based panels on the composition of this cohort. In addition, we aim to describe the origin of the identified variants (de novo or inherited). In addition we identified if different index patients (the first patient in a family in whom the genetic variant is identified) who were independently found to carry the same variant in the GLA-gene were (distantly) related to one another. Identifying a common ancestor can help make the distinction between inherited and de novo mutations, but also result in extra information on life expectancy in ancestors who were obligate carriers.

## Methods

This is an observational longitudinal retrospective study, using data from all patients included in the Dutch national FD database. This database is maintained by the Inherited Metabolic Disease Expert Center of the Amsterdam University Medical Centers, the national referral center for patients with FD in the Netherlands. Patients with FD have been followed and have had their data recorded during outpatient clinic visits since 1999. All individuals included in this database gave written informed consent for use of their data for research purposes (Medical Ethics Committee Amsterdam UMC, CTB number 2014_192). An export of the database used in the analysis of this study was made on December 2^nd^ 2025. Data in the database are pseudonymized and only authorized individuals have access to the key file in which information is linked to identifiable personal data.

Genotype and phenotype data were collected, as well as the year in which DNA analysis was performed, and the indication for and type of genetic test (single gene versus broader DNA sequencing technique). In this analysis we started with inclusion of all patients in our national database with a GLA variant classified as pathogenic or likely pathogenic at the time the diagnostics were performed. For the vast majority of variants, this classification remained unchanged over time [4,5].

The following GLA variants, present in individuals part of our database, are currently classified as benign (not causing Fabry disease) P60L, A143T, D313Y, T385A, W277C and are thus excluded from the current analysis [6]. The R112H variant *is* included in this manuscript, but its pathogenicity has recently become subject of debate [7].

Fabry disease in male patients in our cohort is, irrespective of age, classified either as classical or non-classical based on the presence or absence of classical symptoms, untreated plasma lysoGb3 level and mutation and family history. Male patients are considered to have classical disease if they have one or more of the classical symptoms (cornea verticillata, angiokeratoma and/or acroparesthesia) and a plasma lysoGb3 level of >40 nmol/L [2]. If the untreated plasma lysoGb3 levels were unknown (n = 4), this was extrapolated from a male relative carrying the same variant or the first value under treatment. Fabry disease in male patients that does not meet these criteria is classified as non-classical Fabry disease. Female patients with Fabry disease are divided into two groups: those carrying deleterious variants (leading to classical disease in males) and those with the less deleterious variants (leading to non-classical disease in males).

To investigate the impact of broader DNA analysis methods on phenotypic composition of the Dutch FD cohort, a Chi-squared test was conducted to compare the number of index patients with a classical/severe GLA variant versus those with the non-classical variants and the type of genetic testing performed (single gene vs broad sequencing). Similarly, the number of patients with a classical vs a non-classical variant identified <2011 vs > 2011 were compared. Both NGS and WES/WGS based techniques are included among the broad sequencing techniques irrespective of the indication and content of the gene panel.

The diagnostic laboratories in the Netherlands are accredited according to ISO 15189 (RvA M130) meaning that gene panels are validated before use. In Amsterdam we tested many known variants and found that the sensitivity of our combined test (NextGen and Sanger sequencing) for nucleotide substitutions, deletions, insertions and duplications up to 68 nucleotides is > 99%. In the Netherlands we have agreed that a core gene must have a coverage of >99%. The “core disease genes” in a diagnostic test represent the genes which are considered as essential for establishing a reliable and accurate molecular diagnosis ([6]). The GLA gene is a core gene for hypertrophic cardiomyopathy. If a coding/ splice-junction region has a coverage of <15/30 times (depending on the Dutch laboratory, called low coverage region) that region will be sequenced with Sanger analysis. In Amsterdam the GLA gene always had a good coverage meaning that the GLA gene has a easy to sequence composition (i.e. not very GC or AT rich).

To study if individuals who carry the same GLA variant but were independently diagnosed were distantly related, we constructed pedigrees for all individuals using information from patients themselves regarding the names and dates of birth of parents and grandparents. These pedigrees were extended using birth-, death- and marriage- records from a large number of archiving institutions made publicly available through the website wiewaswie.nl. This website is owned by the CBG, formerly Central Bureau of Genealogy, currently known as the Center for Family History in The Hague and other publicly available sources of genealogical data (e.g., archives of family announcements in newspapers, publicly available pedigrees).

## Results

As of December 2025, the Fabry database includes data on 339 individuals. In one male individual (excluded from the analysis), no variant in the *GLA-gene* was found, although enzyme activity, clinical features and biochemical profile were consistent with a classical phenotype. Two female patients were excluded from the analyses because, although the diagnosis of FD was made, the phenotype (classical vs non-classical) could not conclusively be determined, as there were no male patients having the same genetic variant. In one of these women, the variant was present in mosaic form. Of the remaining 336 individuals, 17 had a benign variant in the *GLA-gene* (not disease causing). They were excluded from the analysis well.

The remaining cohort of 319 individuals with a pathogenic variant in the *GLA-gene* consists of 104 index patients. In our cohort 64 unique GLA variants are present, of which 29 were present in ≤2 individuals. These (likely) pathogenic variants were mostly inherited (n = 275). In 8 cases the variant arose *de novo* (parents were tested and did not carry the mutation, although (non-)paternity was not tested), while in 36 cases this could not be established. The identified variants and associated phenotypes are included in the Fabry working group Genotype Phenotype database, a multicenter open access initiative to provide reliable information on genotype-phenotype associations in FD [8].

**Fig 1:**
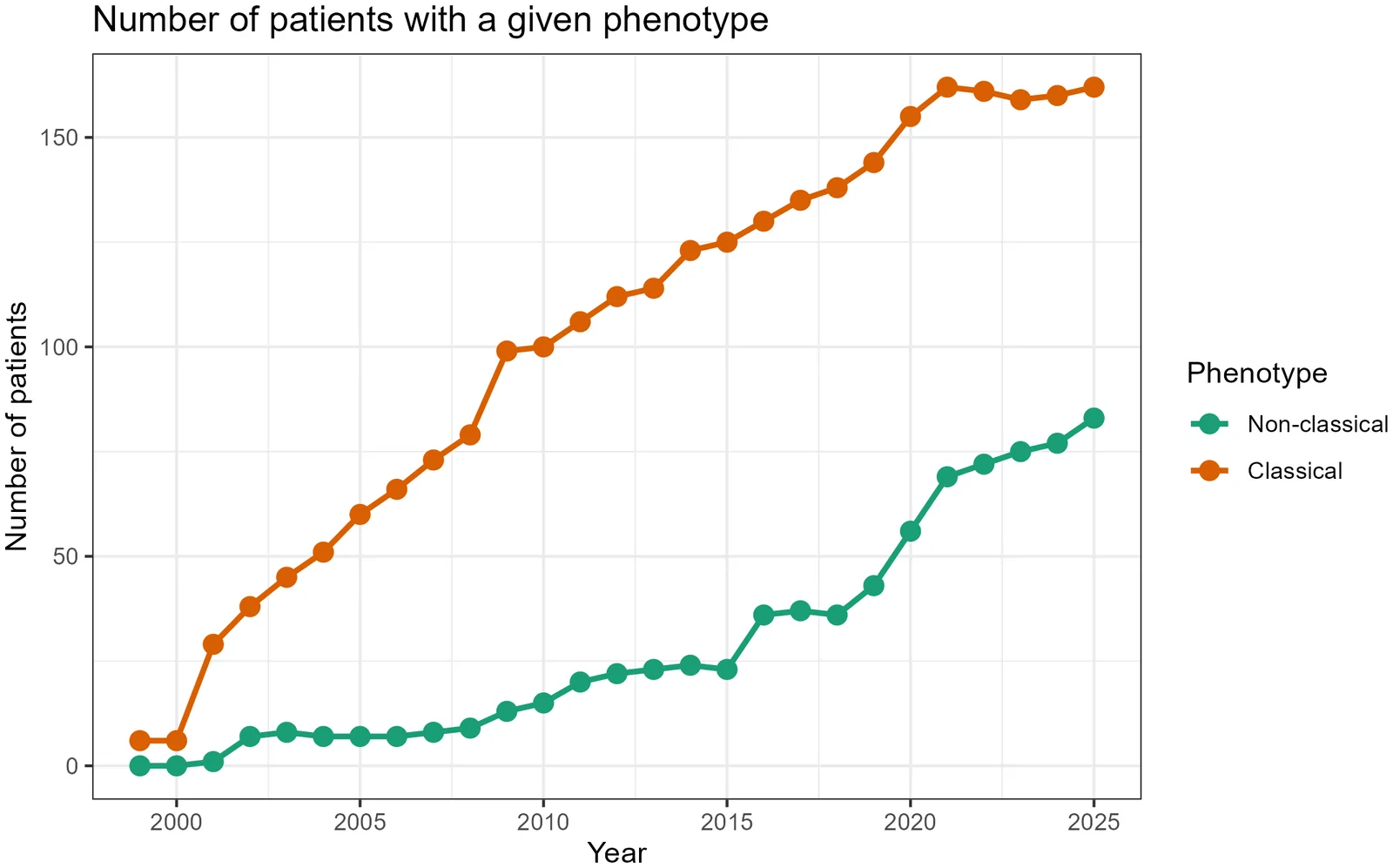
Number of patients with a given phenotype.

Table 1 shows the demographic characteristics of the Amsterdam UMC cohort. A higher proportion of patients with a non-classical GLA variant was diagnosed through broad DNA testing techniques (24%) compared to patients with a classical phenotype (7%) (p < 0.001). When looking only at index patients, 68% of patients with a non-classical GLA variant was diagnosed through broad DNA testing techniques compared to 22% of patients with a classical GLA variant (p < 0.001). More patients with an non-classical variant were identified in recent years (>2011 vs < 2011) compared to patients with a severe/classical variant (p = 0.004). Fig 1 shows the composition of the cohort over time, highlighting the relative increase of patients with attenuated/non-classical variants over time.

**Table 1:** Demographic characteristics of the Amsterdam UMC Fabry disease cohort.

	All		Severe/Classical GLA variant		Attenuated/non-classical GLA variant	
	Male	Female	Male	Female	Male	Female
Number of patients (%)	129 (40)	190 (60)	75 (37)	129 (63)	54 (47%)	61 (53)
Number of index patients	66	38	34	30	32	8
Age, median (range)	48 (13-82)	55 (19-83)	41 (15-66)	56 (18-83)	64 (13-82)	54 (19-81)
Deceased	28	13	16	12	12	1
Targeted analysis of the GLA gene, n (%)	105 (81)	171 (90)	71 (95)	119 (92)	34 (63)	53 (87)
Broad genetic testing, n (%)	24 (19)	18 (10)	4 (5)	10 (8)	20 (37)	8 (13)
Indication for genetic testing, n (%)						
1. Clinical diagnosis prior to possibility of genetic testing	19 (15)	16 (8)	19 (25)	16 (12)	0	0
2. Acroparesthesia/Cornea verticillata/Angiokeratoma	17 (13)	13 (7)	16 (21)	13 (10)	1 (2)	0
3. Cardiomyopathy	25 (19)	17 (9)	3 (4)	11 (9)	22 (41)	6 (10)
4. Renal insufficiency	10 (8)	3 (2)	4 (5)	2 (2)	6 (11)	1 (2)
5. Neurological complications (CVA/TIA)	1 (1)	0	1 (1)	0	0	0
6. Other	7 (5)	4 (2)	3 (4)	3 (2)	4 (7)	1 (2)
7. Family screening	50 (39)	137 (72)	29 (39)	84 (65)	21 (39)	53 (87)

**Fig 2:**
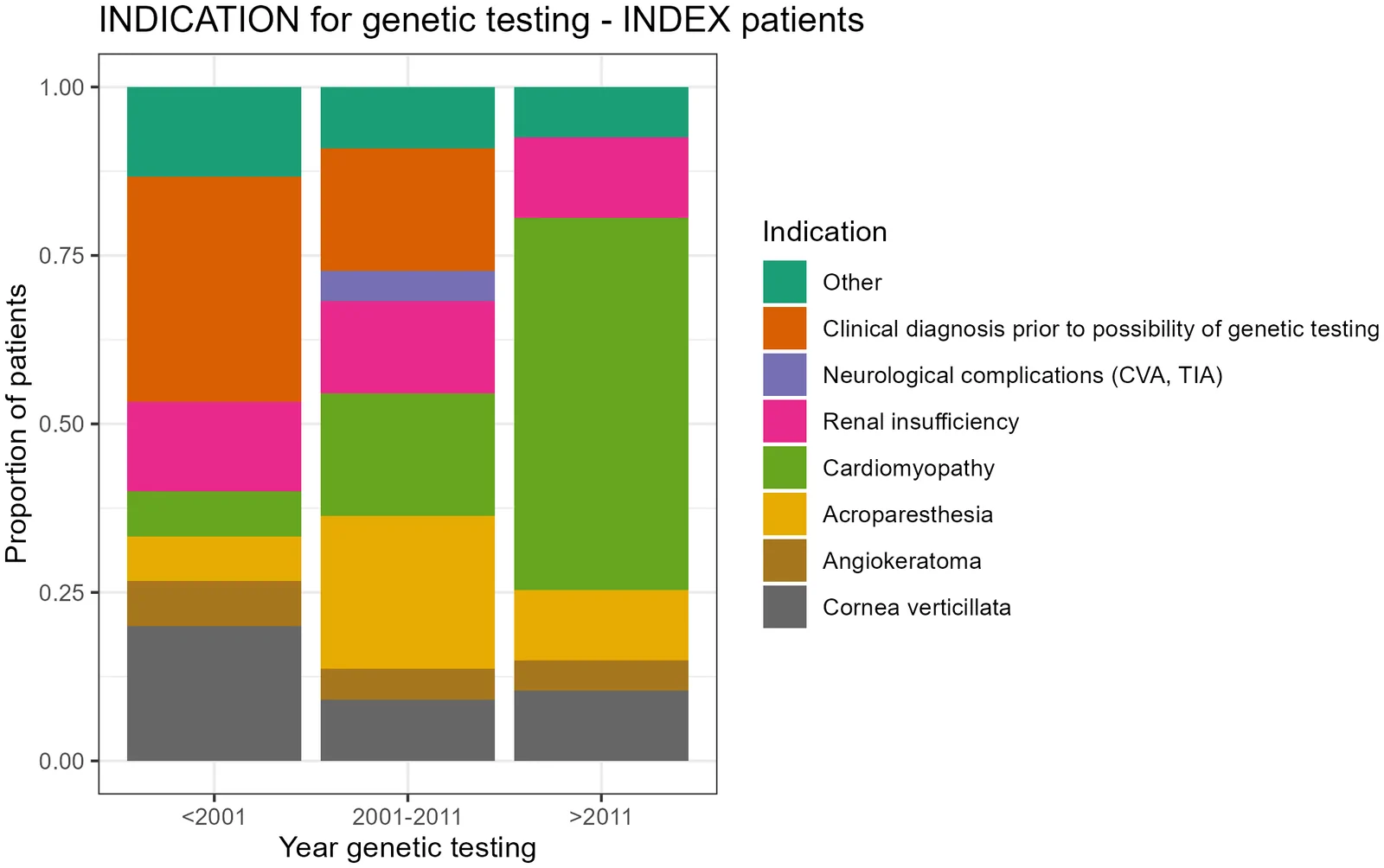
Indications for genetic testing among index patients.

In a subset of families with a classical phenotype, a clinical diagnosis of FD was made prior to the possibility of genetic confirmation of this diagnosis. In these families, the patient in whom the diagnosis was genetically confirmed (the index from a genetic perspective, n = 35) was not always the index patient from a clinical perspective. Of those patients with classical/severe GLA variants in whom the reason for genetic testing could be confirmed (n = 169), the most frequent indication was cascade genetic screening (family screening n = 113, 67%), followed by the presence of classical symptoms (acroparesthesia, cornea verticillata, angiokeratoma)(n = 29, 17%), cardiomyopathy (n = 14, 8%), renal insufficiency (n = 6, 4%), neurological complications (CVA/TIA, n = 1, 1%), or other (n = 6, 4%) reasons. In patients with an attenuated/non-classical variant (n = 115), cascade genetic screening was the most prevalent indication (n = 74, 64%), followed by cardiomyopathy (n = 28, 24%). After the addition of the GLA-gene into NGS panels for HCM, cardiomyopathy became the most important indication for genetic testing among index cases in recent years (Fig 2)

Table 2 shows data on the 6 most common pathogenic GLA variants found in our cohort (I319T, P389A, R342Q, F18S, R220*, R112H). A list of all variants present in our cohort is provided in Table 3. The different variants vary in the ratio of index patients versus the total number of patients carrying the variant. The most common variant (I319T, n = 43) was established independently in 19 patients. This variant is associated with a non-classical phenotype (Veldman et al, manuscript submitted [9]). Genealogical inquiries showed that all individuals in our cohort were (likely) distantly related, sharing a common ancestor from the early 18^th^ century. While some individuals were found to be 4^th^ degree relatives (e.g., first cousins), the degree of kinship was larger for most (max 15^th^ degree). This prompted us to create a fictional pedigree, in an attempt to estimate the number of individuals carrying a I319T GLA variant that might be alive today. This allows a rough estimate of the amount of individuals currently under follow-up versus those that carry the I319T variant, but are not known to have Fabry disease. To this end we used data on the mean number of children per couple and childhood mortality over time, estimating the number of individuals with a I319T GLA variant in a given generation. E.g. a single affected female born in 1850 would have had (on average) 5 children of whom 2 or 3 would have been affected and of whom 2 would have died during childhood. Her 2 affected, surviving children would each have had 5 children of whom 2 or 3 would have been affected etc. Following this method we estimated that a single affected ancestor from 1850 would give rise to approximately 80 affected descendants alive today (6^th^, 7^th^ and 8^th^ generation). Our I319T pedigree identified 4 ancestors born between 1840 and 1860 with 43 identified affected descendants alive today, suggesting that only a minority of the individuals carrying a I319T variant is known to have a risk of developing Fabry disease.

**Table 2:** Details on the 6 most common pathogenic GLA variants found in our cohort.

Mutation c.DNA	Mutation protein level	Associated phenotype	Number of patients	Number of index patients	Number of families
c.956T > C	I319T	Attenuated/non-classical	43	19	1
c.1165C > G	P389A	Attenuated/non-classical	32	6	1
c.1025G > A	R342Q	Severe/classical	24	6	4
c.53T > C	F18S	Severe/classical	17	4	1
c.658C > T	R220*	Severe/classical	17	4	2
c.335G > A	R112H	Attenuated/non-classical – Benign	13	4	2

**Table 3:** Mutations present in the Dutch Fabry cohort and their current associated phenotype.

Mutation, DNA	Mutation, protein level	Phenotype
c.53T > C	F18S	Classical
c.62T > C	L21P	Classical
c.97G > C	D33H	Non-classical
duplication exon 3 + 4		Classical
c.1118G > A	G373D	Classical
c.124A > G	M42V	Classical
c.136C > T	H46Y	Classical
c.157_160delAACC	N53Lfs*57	Classical
c.166delT	C56Afs*65	Classical
c.215T > G	M72R	Classical
c.269G > A	C90Y	Classical
c.316C > T	L106F	Non-classical
c.334C > T	R112C	Classical
c.335G > A	R112H	Non-classical/benign
c.400delT	T134Mfs*31	Classical
c.406G > T	D136Y	Classical
c.422C > T	T141I	Classical
c.436C > T	P146S	Non-classical
c.469del	Q157Rfs*8	Classical
c.548G > A	G183D	Classical
c.551A > G	Y184C	Classical
c.606T > G	C202W	Classical
IVS4 + 1G > A (c.639 + 1G > A)		Classical
IVS4-2A > G (c.640-2A > G)		Classical
c.644A > G	N215S	Non-classical
c.658C > T	R220*	Classical
c.677G > A	W226*	Classical
c.679C > T	R227*	Classical
c.680G > A	R227Q	Classical
c.692A > G	N231G	Classical
c.701A > G	D234G	Classical
c.708G > A	W236*	Non-classical
c.712_719del	S238Efs*9	Classical
c.718_719del	K240Efs*9	Classical
c.728_744del	L243*	Classical
c.779G > A	G260E	Classical
c.792C > A	D264E	Classical
c.793C > T	P265S	Classical
c.803T > C	L268S	Classical
c.893A > G	N298S	Non-classical
c.897C > G	D299E	Non-classical
c.898C > T	L300F	Classical
c.901C > T	R301*	Classical
c.908_910delTCA	I303del	Classical
c.955_969delinsTTGC	I319Lfs*10	Classical
c.956T > C	I319T	Non-classical
c.963_964delinsCA	Q321_D322delinsHN	Classical
c.996_999del	Q333Efs*14	Classical
c.997C > T	Q333*	Classical
IVS6-2A > T (c.1000-2A > T)		Classical
c.1021G > A	E341K	Classical
c.1025G > A	R342Q	Classical
c.1040dupT	L347Ffs*28	Classical
c.1054_1062del	A352_I354del	Classical
c.1072G > C	E358Q	Non-classical
c.1073A > G	E358G	Classical
c.1074_1075del	E358Dfs*16	Classical
c.1088G > A	R363H	Non-classical
c.1094dup	Y365*	Classical
c.1107del	A370Lfs*21	Classical
c.1124_1176del	G375Efs	Classical
c.1156C > T	Q386*	Classical
c.1165C > G	P389A	Non-classical
c.1246C > T	Q416*	Classical

For the P389A and F18S variants a single common ancestor was identified as well. While for the other variants some individuals proved to be distantly related, not all individuals could be traced back to one common ancestor. While the above mentioned 6 variants represent the most common variants in the Dutch cohort, they have been described in individuals from other countries as well and are thus not unique for the Dutch population.

**Fig 3:**
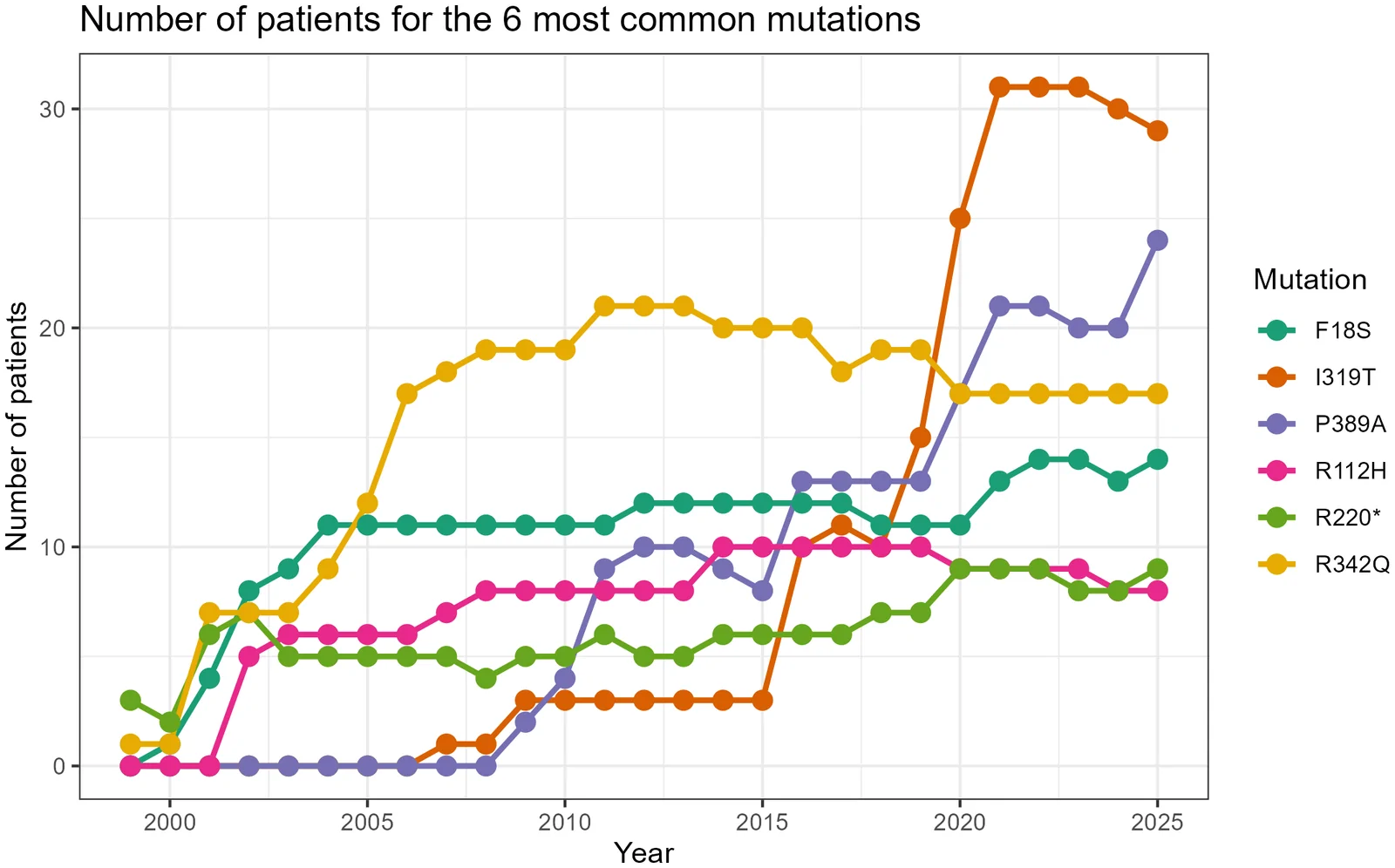
Number of patients for the 6 most common mutations.

Fig 3 shows the number of patients per year for the 6 most common variants. Of particular interest is the fact that the number of patients carrying the I319T mutation shows a significant increase since 2011, which is after introduction of the broader sequencing techniques.

## Discussion

The current study confirms that Fabry disease is a genetically heterogeneous disorder with 64 different *GLA* variants established in a cohort of 319 patients. The *GLA* variants are mostly inherited, with *de novo* occurrence found in a minority of patients. This study shows that the introduction of broader DNA sequencing techniques and subsequent addition of the GLA-gene to various NGS or WES based panels has a significant impact on the type of GLA variants that are identified and subsequently on the phenotypic composition of the cohort as GLA-sequencing performed in a broader range of individuals with less specific symptoms.

Generally speaking, single gene analysis was performed until the early/mid-2010s in individuals with a specific clinical suspicion of FD. This identified a high number of patients carrying high impact GLA variants leading to classical FD in male patients and cardiomyopathy in female patients, who were mainly diagnosed through family screening. The fact that genetic testing at that time took longer and costs of consecutive Sanger sequencing of multiple candidate genes for (for instance) cardiomyopathy were higher will have contributed to the fact that these tests were performed in individuals with a high a priori chance of a Fabry disease diagnosis. With the introduction of panel analysis (NGS or WES based) and addition of the GLA-gene to various panels, diagnostics were performed in individuals with less specific clinical symptoms (e.g., isolated hypertrophic cardiomyopathy). This led to identification of less disruptive GLA variants in individuals with GLA insufficiency (but not deficiency), lower substrate ((lyso)Gb3) levels and later onset of symptoms and complications. Moreover, for some variants their pathogenicity can remain topic of debate for decades creating uncertainty for individuals that carry them [7,10,11].

The shift in phenotypic composition as a result of the addition of the GLA-gene to NGS or WES based panels should not come as a surprise, as the attenuated phenotype of FD is more difficult to recognize clinically. The broadening of the phenotypical spectrum is not unique for FD, but is also observed in many other disorders (e.g., [12]. Broader DNA sequencing techniques have resulted in a correct diagnosis made in numerous individuals in whom an explanation for their symptoms would not otherwise have been found. This is highly valuable both from an individual as well as family perspective.

It is nonetheless important to recognize this shift as this should be taken into account when interpreting prior research in FD. FD specific treatment has been available since 1999 in the form of enzyme replacement therapy. Early clinical trials have thus been performed in cohorts that consisted mostly of patients with a classical phenotype. Comparing, for instance, the occurrence of cardiac-, neurological or renal complications among patients in these early clinical trials to the rate in patients in current trials would lead to a bias in favor of current trials, as complication rates are lower in patients with an attenuated/non-classical phenotype. In addition, individuals with less disruptive GLA variants require less stringent monitoring (especially in childhood) and a different treatment approach (Veldman et al, submitted [9]).

Genealogical research revealed that patients carrying the same GLA-variant were often (distantly) related. Pedigrees for the different variants reveal a number of ancestors as obligate carriers of the disease causing variant. While medical records are obviously not available for these ancestors, their age at death could be extracted from publicly available birth- and death records, shedding light on the impact of the variant on life expectancy. These data will be further explored in additional analysis of standardized mortality ratios (manuscript in preparation).

Pedigree analysis reveals that the diagnosis of FD has not been made in a high proportion of individuals identified as obligate carriers. Furthermore, a significant number of descendants of these carriers are alive today. Age of onset and phenotype of FD are such that reproductive fitness is not affected so this does not explain these low numbers, but several explanations exist. To begin with, relatives may be aware of their risk, but may choose not to undergo genetic testing. For instance, the uptake of genetic testing was approximately 60% in a study of relatives of patients with inherited cardiac conditions [13]. Given the high number of index patients in our I319T-cohort, the high number of ‘missing’ affected relatives cannot (fully) be explained by the informed choice not to be tested for the familial mutation.

Alternatively, if these individuals are unaware of their risk, two explanations remain. Either these family members *do* suffer from FD related symptoms and complications, but the diagnosis is not recognized. *Or*, these family members might be less severely affected and never develop complications requiring medical care. In the latter case, these family members are rightfully missed; a ‘patient’ who does not suffer from any signs or symptoms should not be labeled as a patient and might be harmed if we do so. This is especially true for females carrying less severe GLA variants, as we generally do not observe clinical symptoms in these women [3]. In our view, these women should receive adequate counseling regarding the possibility to test for FD in their male offspring once they reach adulthood, but they do not need FD specific treatment nor frequent follow-up.

On the other hand, for the patients in the first scenario, the opportunity to start the correct follow-up and (concomitant) treatment is missed. Active cascade genetic screening could be an important tool in trying to identify all affected family members, but the justification for this approach should be based on the expected severity of the phenotype associated with a specific variant. Genetic counseling and ensuing follow-up for FD in metabolic clinics should be tailored to individual GLA-variants.

The proposed GLA-variant specific tailored approach should also be reflected in initiatives to include Fabry disease in new born screening programs (NBS). NBS programs are aimed at early detection of serious but treatable conditions in which early treatment is beneficial, so that a disorder can be treated timely and the outcome for patients is improved compared to the situation in which the diagnosis was made via the regular route. GLA-variants causing a non-classical FD phenotype do not meet these criteria; age of onset of signs and symptoms in males is far beyond the childhood years and women may not develop symptoms at all ([3,14] and these individuals will thus not benefit from early treatment with FD specific therapy.

FD has been included in various NBS programs worldwide since 2006 [15], but might identify large numbers of individuals with GLA-variants associated with non-classical disease, variants of unknown significance or even benign variants. For instance, a recently published study by Paltzer et al reports on 88 individuals referred with abnormal NBS for Fabry. Of those 88 patients, only 6 had a GLA variant associated with classical FD [16]. What’s more, the majority of patients (n = 31, 35%) in their cohort carried the A143T variant. This variant has been the subject of much debate, but is regarded by us and many others as a benign variant [17]. Paltzer et al expanded their cohort identified by NBS by subsequent cascade testing to a cohort of 80 individuals carrying the A143T variant. None of the individuals identified had a significant elevation of lysoGb3 and none of the individuals have had Fabry related signs or symptoms. In our opinion, NBS for Fabry disease, *if* considered, should be primarily targeted at finding male patients with a classical phenotype as those patients would benefit from an early diagnosis. This may require filtering NBS results for variants known to be associated with the classical phenotype and/or incorporating lysoGb3 results in the screening algorithm. Given the high number of individuals with variants of less/limited actionability, NBS in its current form for FD might do more harm than good. Alternatively, there might be much to win in terms of getting the proper care and follow-up in place for individuals by applying more active cascade screening for actionable variants.

In conclusion, this study confirms that Fabry disease is a genetically and phenotypically heterogeneous disorder and shows that the introduction of broader DNA sequencing techniques and subsequent addition of the GLA-gene to NGS or WES panels resulted in new diagnoses in individuals in whom symptoms would have otherwise remained unexplained, but also in a gradual shift in the phenotypic composition of Fabry disease patient cohorts. This has important consequences when comparing current to historical clinical and treatment effect data and requires GLA-variant specific tailored genetic counseling and clinical follow-up.
