# Supplementary material for: The changing landscape of Fabry disease: Impact of the inclusion of the GLA-gene in broader NGS or WES based panels on the phenotypic spectrum
Source: PLoS One. 2026 Sep 16;21(9):e0358572. doi: 10.1371/journal.pone.0358572 (PMC13581027; doi:10.1371/journal.pone.0358572)
Supplement: Supplementary file 1 [file pone.0358572.s002.pdf]

May 28, 2026

Dear editor, dear reviewers,

We thank the reviewers for their valuable feedback and hope to have addressed the issues they raised satisfactory. In the text below, we have addressed their comments point by point. We feel confident that the manuscript has improved substantially.

On behalf of the co-authors,
Sincerely,
Laura van Dussen, MD PhD
Prof. Mirjam Langeveld, MD PhD

Data Availability Statement

The manuscript and its Supporting Information files contain all relevant data.
Both ethical and legal restriction prevent us from submitting our full raw data set. The dataset contains privacy sensitive information such as mutation, year of birth and gender which, given the rarity of Fabry disease, may lead to identification of individual patients despite being pseudonymized. We promote inclusive and collaborate research and anyone wishing to collaborate with us could contact the last author and, provided all conditions are met and a data sharing agreement is in place, raw data can be made available.

1. Is the manuscript technically sound, and do the data support the conclusions?

The manuscript must describe a technically sound piece of scientific research with data that supports the conclusions. Experiments must have been conducted rigorously, with appropriate controls, replication, and sample sizes. The conclusions must be drawn appropriately based on the data presented.
Reviewer #1: Yes
Reviewer #2: Partly

---

2. Has the statistical analysis been performed appropriately and rigorously?
Reviewer #1: N/A
Reviewer #2: No

---

3. Have the authors made all data underlying the findings in their manuscript fully available?

The PLOS Data policy requires authors to make all data underlying the findings described in their manuscript fully available without restriction, with rare exception (please refer to the Data Availability Statement in the manuscript PDF file). The data should be provided as part of the manuscript or its supporting information, or deposited to a public repository. For example, in addition to summary statistics, the data points behind means, medians and variance measures should be available. If there

are restrictions on publicly sharing data—e.g. participant privacy or use of data from a third party—those must be specified.
Reviewer #1: Yes
Reviewer #2: No

---

4. Is the manuscript presented in an intelligible fashion and written in standard English?

PLOS ONE does not copyedit accepted manuscripts, so the language in submitted articles must be clear, correct, and unambiguous. Any typographical or grammatical errors should be corrected at revision, so please note any specific errors here.
Reviewer #1: Yes
Reviewer #2: No

---

5. Review Comments to the Author

Please use the space provided to explain your answers to the questions above. You may also include additional comments for the author, including concerns about dual publication, research ethics, or publication ethics. (Please upload your review as an attachment if it exceeds 20,000 characters)

Reviewer #1: Van Dussen et al presents the type of variants and ascertain spectrum of the Dutch Fabry disease cohort from 1999 until now, and how the spectrum of pathogenic variants unsurprisingly have shifted from mainly the ones associated with classical Fabry disease to mainly the ones associated with non-classical disease, foremost cardiomyopathy. It is interesting to know the de novo variant frequency from such a large and well-characterized cohort (8 in 319), and that many variants have likely founders (and a lot of unascertained individuals).
The article illustrates the challenge when moving from a monogenic cause of a disease (classical Fabry variants) to an oligofactorial/multifactorial disease spectrum where GLA variants is probably just a contributing part to the story, and the size of this contribution is largely unknown (but it could be major). I like their recommendation to base patient follow-up (and counselling) also on the genotype.
Major objections:
1. It is not the broader sequencing techniques that is the cause of the above-mentioned shift, but changed variant ascertainment. In theory, Sanger sequencing could have found the milder variants just as well as NGS, but since the GLA gene is included in gene panels (like for HCM), other groups of patients will get GLA variants discovered. The authors know this, but emphasis “broader sequencing techniques” as the explanation. I think it is the inclusion of GLA in gene panels that is the main explanation, i.e. GLA variant testing on weaker indications than before.

**<u>Response</u>**
The reviewer is absolutely right in stating that it is not the introduction of the broader sequencing techniques perse, but rather the subsequent addition of the GLA-gene to the various gene panels and

WES filters that caused the phenotypic shift. We have amended the title and text to reflect this shift in interpretation as this is what we meant to say.

**The title was changed to:**
*The changing landscape of Fabry disease: impact of the inclusion of the GLA-gene in broader NGS or WES based panels on the phenotypic spectrum*

**In the abstract we added:**
In this study we investigated the impact of the introduction of broader DNA sequencing techniques *and subsequently the addition of the GLA-gene to NGS or WES based panels* on the type of FD patient that is diagnosed, by analyzing changes in the composition of the Dutch Fabry cohort over time.

**We have added the following sentence to our introduction:**
*Following the introduction of these techniques, the GLA-gene has been added to several NGS or WES based panels such as (hypertrophic) cardiomyopathy, renal disorders and polyneuropathies.*

**In the following paragraph we added:**
The objective of this article is to describe the genetic and phenotypic heterogeneity in a large, single country FD cohort and to study the impact of the introduction of broader DNA sequencing techniques *and subsequent inclusion of the GLA-gene in several NGS or WES based panels* on the composition of this cohort.

**In the discussion we amended the first paragraph:**
The current study confirms that Fabry disease is a genetically heterogeneous disorder with 64 different *GLA* variants established in a cohort of 319 patients. The *GLA* variants are mostly inherited, with *de novo* occurrence found in a minority of patients. This study shows that the introduction of broader DNA sequencing techniques *and subsequent addition of the GLA-gene to various NGS or WES based panels* has a significant impact on the type of GLA variants that are identified and subsequently on the phenotypic composition of the cohort *as GLA-sequencing is performed in a broader range of individuals with less specific symptoms.*

**And added the following sentence:**
With the introduction of panel analysis (NGS or WES based) *and addition of the GLA-gene to various panels*, diagnostics were performed in individuals with less specific clinical symptoms (e.g. isolated hypertrophic cardiomyopathy).
The shift in phenotypic composition as a result *of the addition of the GLA-gene to NGS or WES based panels* should not come as a surprise

**In the conclusion we added the following sentence:**

In conclusion, this study confirms that Fabry disease is a genetically and phenotypically heterogeneous disorder and shows that the introduction of broader DNA sequencing techniques *and subsequent addition of the GLA-gene to NGS or WES panels* resulted in new diagnoses in individuals in whom symptoms would have otherwise remained unexplained, but also in a gradual shift in the phenotypic composition of Fabry disease patient cohorts.

Minor comments:

1. “Neuronopathic pain” is rarely used, I prefer “neuropathic pain”.

__**Response**__

We changed this.

2. Sanger sequencing was not more expensive or took longer time than today’s NGS-based testing (GLA is a small gene that can be fully sequencing after a small number of amplifications).

__**Response**__

We agree that targeted Sanger sequencing of just the GLA-gene would not have been more expensive than running a single NGS based panel for cardiomyopathies. However, before panel diagnostics became available, in patients with an isolated cardiomyopathy, the GLA-gene would not necessarily have been the first candidate gene for targeted sequencing. More likely, other genes such as MYBPC3 and MYH7 would have been sequenced first before Sanger sequencing of GLA would be considered. The costs of sequencing multiple genes consecutively by Sanger sequencing at that time would have been higher than those of an NGS based panel test today. **We amended the text as follows:**

The fact that genetic testing at that time took longer and costs *of consecutive Sanger sequencing of multiple candidate genes for (for instance) cardiomyopathy* were higher will have contributed to the fact that these tests were performed in individuals with a high a priory chance of a Fabry disease diagnosis.

3. “GLA insufficiency” is mentioned in the discussion, but the reference to the article recommending the use of this term (instead of non-classical Fabry disease) is not included (even though the last author was a co-author on this recommendation).

__**Response**__

We have added the following suggested reference to the article

Houge G, Langeveld M, Oliveira JP. GLA insufficiency should not be called Fabry disease. Eur J Hum Genet. 2025;33(3):263–5.

4. The article can be shortened without loss of new information content.

__**Response**__

We appreciate the suggestion and have attempted to shorten the manuscript.

Reviewer #2: Revision of the manuscript: “The changing landscape of Fabry disease; impact of the use of broad sequencing techniques on GLA variant detection and the associated phenotypes”
This manuscript presents an observational, longitudinal retrospective analysis of the Dutch national Fabry disease (FD) cohort, aiming to evaluate how the introduction of broader sequencing techniques has influenced genotype detection and phenotypic distribution. The topic is relevant and timely, particularly given the increasing use of next-generation sequencing (NGS) in clinical genetics. The dataset is valuable, and the longitudinal nature of the cohort (since 1999) represents a strength. However, the manuscript requires substantial revision before it can be considered for publication. Major concerns relate to language quality, methodological clarity, data interpretation, and presentation of results (tables and figures). Additionally, some conclusions are speculative and insufficiently supported by data.

Major Comments
1.Language and Editorial Quality
The manuscript contains numerous grammatical, syntactical, and orthographic errors that significantly impair readability. Examples include inconsistent terminology (e.g., “alfa-galactosidase” vs “alpha-galactosidase”), incorrect punctuation (notably the use of “;” in the title), and spelling mistakes such as “arrhythmias” and “a priori.” A comprehensive English language revision by a professional editor or native speaker is strongly recommended. This should include standardization of terminology and correction of sentence structure throughout the manuscript.

**<u>Response</u>**
We have carefully checked our manuscript and made a number of corrections throughout the manuscript that we hope improve the readability.

2.Definition of Diagnostic Criteria
The diagnostic criteria for FD, particularly the lyso-Gb3 cut-off values, are unclear and inconsistently presented. The authors should:
• Clearly justify each cut-off value
• Reference established guidelines or prior studies supporting these thresholds
• Explain how these values were applied consistently across the cohort

**<u>Response</u>**
The reviewer is right in pointing out that our use of the cut-off values for lysoGb3 is inconsistent and we have amended this in the manuscript (see below).
This is a longitudinal cohort followed over more than 20 years. During this follow-up lysoGb3 was introduced as a biomarker (2008) and subsequently became part of the follow-up of our patients and interpretation of the pathogenicity of GLA-variants.
The pathogenicity of several variants, such as the P60L variant, was reclassified from pathogenic to benign because of evolving insight. This is a continuous process. A recent example is the R112H variant. Initially classified as pathogenic and associated with a non-classical phenotype, our current

understanding is that this variant causes GLA-insufficiency but not Fabry disease (9), though this opinion will not be shared with all Fabry researchers and clinicians. Given the discussion on this variant, which is a good example of the debate on monogenetic disease vs an oligofactorial/multifactorial disease spectrum, we did include patients with this variant in the current paper, but added extra explanation with a reference to the publication. A full list of all genetic variants present in our cohort and their current classification is additionally provided in table 3.

**Our amended methods section now reads as follows:**
In this analysis we started with inclusion of all patients in our national database with a GLA variant classified as pathogenic or likely pathogenic at the time the diagnostics were performed. For the vast majority of variants, this classification remained unchanged over time. The following GLA variants, present in individuals part of our database, are currently classified as benign (not causing Fabry disease) P60L, A143T, D313Y, T385A, W277C and are thus excluded from the current analysis (6). The R112H variant *is* included in this manuscript, but its pathogenicity has recently become subject of debate (9).

Fabry disease in male patients in our cohort is, irrespective of age, classified either as classical or non-classical based on the presence or absence of classical symptoms, untreated plasma lysoGb3 level and GLA variant and family history. Male patients are considered to have classical disease if they have: one or more of the classical symptoms (cornea verticillata, angiokeratoma and/or acroparesthesia) and a plasma lysoGb3 level of >40 nmol/L *(2)*. If the untreated plasma lysoGb3 levels were unknown (n=4), this was extrapolated from a male relative carrying the same variant or the first value under treatment. Fabry disease in male patients that does not meet these criteria is classified as non-classical Fabry disease. Female patients with Fabry disease are divided into two groups: those carrying deleterious variants (leading to classical disease in males) and those with the less deleterious variants (leading to non-classical disease in males).

3.Genetic Testing Methodology
The description of "broad sequencing techniques" lacks technical detail. Critical information is missing, including:
• Type of panels used (gene content)
• Coverage metrics (depth, sensitivity)
• Bioinformatics pipeline and variant filtering criteria

<u>**Response**</u>
We agree with the reviewer that this is relevant information. The Amsterdam UMC is the national referral center for patients with Fabry disease in the Netherlands and patients are referred to here from hospitals throughout the country, often after genetic testing was performed and a GLA-variant was identified. Genetic testing has been performed in multiple centers and over >10 years since the introduction of NGS (and subsequently WES) based panels. Considering the retrospective nature of this observational, longitudinal analysis, it is not possible to obtain the gene content, coverage metrics and bioinformatics pipelines used over time .

The diagnostic labs in the Netherlands are accredited according to ISO 15189 (RvA M130) meaning that gene panels are validated before use. In Amsterdam we tested many known variants and found that the sensitivity of our combined test (NextGen and Sanger sequencing) for nucleotide substitutions, deletions, insertions and duplications up to 68 nucleotides is >99%. In the Netherlands we have agreed that a core gene must have a coverage of >99%. The "core disease genes" in a diagnostic test represent the genes which are considered as essential for establishing a reliable and accurate molecular diagnosis (see Weiss MM et al, 2013, Human Mutat, 34:1313-1321). The GLA gene is a core gene for hypertrophic cardiomyopathy. If a coding/ splice-junction region has a coverage of <15/30 times (depending on the Dutch laboratory, called low coverage region) that region will be sequenced with Sanger analysis. In Amsterdam the GLA gene always had a good coverage meaning that the GLA gene has a easy to sequence composition (i.e. not very GC or AT rich).

**We have added the following explanation to our methods section:**
Both NGS and WES/WGS based techniques are included among the broad sequencing techniques irrespective of the indication and content of the gene panel.
The diagnostic laboratories in the Netherlands are accredited according to ISO 15189 (RvA M130) meaning that gene panels are validated before use. In Amsterdam we tested many known variants and found that the sensitivity of our combined test (NextGen and Sanger sequencing) for nucleotide substitutions, deletions, insertions and duplications up to 68 nucleotides is >99%. In the Netherlands we have agreed that a core gene must have a coverage of >99%. The "core disease genes" in a diagnostic test represent the genes which are considered as essential for establishing a reliable and accurate molecular diagnosis (see Weiss MM et al, 2013, Human Mutat, 34:1313-1321). The GLA gene is a core gene for hypertrophic cardiomyopathy. If a coding/ splice-junction region has a coverage of <15/30 times (depending on the Dutch laboratory, called low coverage region) that region will be sequenced with Sanger analysis. In Amsterdam the GLA gene always had a good coverage meaning that the GLA gene has a easy to sequence composition (i.e. not very GC or AT rich).

4.Results and Data Presentation
Tables and figures are incomplete and inadequately described. Specifically:
• Tables lack proper titles and explanatory legends
• Figures (e.g., Figures 1–3) are presented without sufficient captions or interpretation
• Axes, units, and statistical annotations are not clearly explained
For example, the trend shown in Figure 1 (page 20) demonstrates an increase in non-classical phenotypes over time, but no quantitative analysis of this increase is provided in the text.
All tables and figures must be:
• Fully labeled
• Referenced appropriately in the text
• Accompanied by clear legends explaining the data

<u>**Response**</u>
We have added titles for table 1 and 2 and amended the axes titles and legend to explain the figures more clearly. All tables and figures are referenced in the text.

The reviewer correctly states that no quantitative analysis of the increase in the number of patients with a non-classical phenotype over time, as depicted in figure 1, is provided in the text. We did perform a formal analysis, but did not include the result in the manuscript.

**We have now added the analysis in the text:**

To investigate the impact of broader DNA analysis methods on phenotypic composition of the Dutch FD cohort, a Chi-squared test was conducted to compare the number of index patients with a classical/severe GLA variant versus those with the non-classical variants and the type of genetic testing performed (single gene vs broad sequencing). *Similarly, the number of patients with a classical vs a non-classical variant identified <2011 vs >2011 were compared. Both NGS and WES/WGS based techniques are included among the broad sequencing techniques irrespective of the indication and content of the gene panel.*

*More patients with an non-classical variant were identified in recent years (>2011 vs <2011) compared to patients with a severe/classical variant (p=0.004) .* Figure 1 shows the composition of the cohort over time, highlighting the relative increase of patients with attenuated/nonclassical variants over time.

5. Extrapolation of Undiagnosed Cases
The extrapolation of the number of undiagnosed individuals based on genealogical modeling (e.g., estimation from the I319T variant pedigree) is highly speculative and insufficiently validated.
While the concept of a founder effect is relevant, the current analysis lacks:
• Robust statistical modeling
• Sensitivity analyses
• Comparison with established population-based studies
The authors should either:
• Substantially strengthen this section with rigorous methodology, or
• Reduce speculative claims and present it as a hypothesis-generating observation
The work by Azevedo et al. (2020) on founder effects is the example of that research

**Response**
We thank the reviewer for this suggestions. We have now adapted this section to better support our hypothesis.

This prompted us to *create* a fictional pedigree, *in an attempt to estimate* the number of individuals carrying a I319T GLA variant that *might* be alive today. This allows a rough estimate of the amount of individuals currently under follow-up versus those that carry the I319T variant but are not known to have Fabry disease. To this end we used data on the mean number of children per couple and childhood mortality over time, estimating the number of individuals with a I319T GLA variant in a given generation. E.g. a single affected female born in 1850 would have had (on average) 5 children of whom 2 or 3 would have been affected and of whom 2 would have died during childhood. Her 2 affected, surviving children would each have had 5 children of whom 2 or 3 would have been affected

etc. Following this method we estimated that a single affected ancestor from 1850 would give rise to approximately 80 affected descendants alive today (6th, 7th and 8th generation). Our I319T pedigree identified 4 ancestors born between 1840 and 1860 with 43 identified affected descendants alive today, *suggesting that only a minority* of the individuals carrying a I319T variant is known to have a risk of developing Fabry disease .

Conclusion
This manuscript addresses an important and evolving aspect of Fabry disease diagnostics, highlighting the impact of broad sequencing techniques on phenotypic classification. However, major revisions are required to improve language quality, methodological transparency, data presentation, and scientific rigor.
In its current form, the manuscript does not yet meet the standards required for publication, but with substantial revision, it has the potential to contribute meaningfully to the field.

**Response**
We thank the reviewer for their valuable feedback and hope to have successfully addressed the issues raised.

---

6. PLOS authors have the option to publish the peer review history of their article (what does this mean?). If published, this will include your full peer review and any attached files.

If you choose "no", your identity will remain anonymous but your review may still be made public.

**Do you want your identity to be public for this peer review?** For information about this choice, including consent withdrawal, please see our Privacy Policy.
Reviewer #1: No
Reviewer #2: No
