# Supplementary material for: The changing landscape of Fabry disease: Impact of the inclusion of the GLA-gene in broader NGS or WES based panels on the phenotypic spectrum
Source: PLoS One. 2026 Sep 16;21(9):e0358572. doi: 10.1371/journal.pone.0358572 (PMC13581027; doi:10.1371/journal.pone.0358572)
Supplement: Peer review history

# Author comment: Author response to Decision Letter 0

**DOI:** 10.1371/journal.pone.0358572.r001

**Transfer Alert**

This paper was transferred from another journal. As a result, its full editorial history (including decision letters, peer reviews and author responses) may not be present.

17 Feb 2026

# Peer review: Decision Letter 0

**DOI:** 10.1371/journal.pone.0358572.r002

**Authors:** Maria de Fátima Macedo

22 Apr 2026

Dear Dr. van Dussen,

Thank you for submitting your manuscript to PLOS ONE. After careful consideration, we feel that it has merit but does not fully meet PLOS ONE’s publication criteria as it currently stands. Therefore, we invite you to submit a revised version of the manuscript that addresses the points raised during the review process.

Please submit your revised manuscript by Jun 06 2026 11:59PM. If you will need more time than this to complete your revisions, please reply to this message or contact the journal office at plosone@plos.org. When you're ready to submit your revision, log on to https://www.editorialmanager.com/pone/ and select the 'Submissions Needing Revision' folder to locate your manuscript file.

- A letter that responds to each point raised by the reviewers. You should upload this letter as a separate file labeled 'Response to Reviewers'.
- A marked-up copy of your manuscript that highlights changes made to the original version. You should upload this as a separate file labeled 'Revised Manuscript with Track Changes'.
- An unmarked version of your revised paper without tracked changes. You should upload this as a separate file labeled 'Manuscript'.

If you would like to make changes to your financial disclosure, please include your updated statement in your cover letter. Guidelines for resubmitting your figure files are available below the reviewer comments at the end of this letter.

If applicable, we recommend that you deposit your laboratory protocols in protocols.io to enhance the reproducibility of your results. Protocols.io assigns your protocol its own identifier (DOI) so that it can be cited independently in the future. For instructions see: https://journals.plos.org/plosone/s/submission-guidelines#loc-laboratory-protocols. Additionally, PLOS ONE offers an option for publishing peer-reviewed Lab Protocol articles, which describe protocols hosted on protocols.io. Read more information on sharing protocols at https://plos.org/protocols?utm_medium=editorial-email&utm_source=authorletters&utm_campaign=protocols.

As the corresponding author, your ORCID iD is verified in the submission system and will appear in the published article. PLOS supports the use of ORCID, and we encourage all coauthors to register for an ORCID iD and use it as well. Please encourage your coauthors to verify their ORCID iD within the submission system before final acceptance, as unverified ORCID iDs will not appear in the published article. *Only*  the individual author can complete the verification step; PLOS staff *cannot*  verify ORCID iDs on behalf of authors.

We look forward to receiving your revised manuscript.

Kind regards,

Fátima Macedo, Ph.D.

Academic Editor

PLOS One

Journal Requirements:

When submitting your revision, we need you to address these additional requirements.

1. Please ensure that your manuscript meets PLOS ONE's style requirements, including those for file naming. The PLOS ONE style templates can be found at

https://journals.plos.org/plosone/s/file?id=wjVg/PLOSOne_formatting_sample_main_body.pdf and

https://journals.plos.org/plosone/s/file?id=ba62/PLOSOne_formatting_sample_title_authors_affiliations.pdf

2. In your ethics statement, please ensure you have provided the full name of the ethics committee(s) that approved this study. If more than 5 committees provided approval, it is acceptable to provide a list as a Supporting Information file.

3. Please provide additional details regarding participant consent. In the ethics statement in the Methods and online submission information, please ensure that you have specified (1) whether consent was informed and (2) what type you obtained (for instance, written or verbal, and if verbal, how it was documented and witnessed). If your study included minors, state whether you obtained consent from parents or guardians. If the need for consent was waived by the ethics committee, please include this information.

If you are reporting a retrospective study of medical records or archived samples, please ensure that you have discussed whether all data were fully anonymized before you accessed them and/or whether the IRB or ethics committee waived the requirement for informed consent. If patients provided informed written consent to have data from their medical records used in research, please include this information.

4. Please update your submission to use the PLOS LaTeX template. The template and more information on our requirements for LaTeX submissions can be found at http://journals.plos.org/plosone/s/latex.

5. Thank you for stating the following in the Competing Interests section: [Mirjam Langeveld is involved in pre‐marketing studies with Sanofi and Chiesi. AMC Research BV manages all financial arrangements. No fees, travel reimbursements, subsidies or grants, related to the presented work or otherwise, have been obtained from pharmaceutical companies.].

Please confirm that this does not alter your adherence to all PLOS ONE policies on sharing data and materials, by including the following statement: ""This does not alter our adherence to  PLOS ONE policies on sharing data and materials.” (as detailed online in our guide for authors http://journals.plos.org/plosone/s/competing-interests).  If there are restrictions on sharing of data and/or materials, please state these. Please note that we cannot proceed with consideration of your article until this information has been declared.

Please include your updated Competing Interests statement in your cover letter; we will change the online submission form on your behalf.

6. We note that your Data Availability Statement is currently as follows: [All relevant data are within the manuscript and its Supporting Information files]

Please confirm at this time whether or not your submission contains all raw data required to replicate the results of your study. Authors must share the “minimal data set” for their submission. PLOS defines the minimal data set to consist of the data required to replicate all study findings reported in the article, as well as related metadata and methods (https://journals.plos.org/plosone/s/data-availability#loc-minimal-data-set-definition).

For example, authors should submit the following data:

- The values behind the means, standard deviations and other measures reported;

- The values used to build graphs;

- The points extracted from images for analysis.

Authors do not need to submit their entire data set if only a portion of the data was used in the reported study.

If your submission does not contain these data, please either upload them as Supporting Information files or deposit them to a stable, public repository and provide us with the relevant URLs, DOIs, or accession numbers. For a list of recommended repositories, please see https://journals.plos.org/plosone/s/recommended-repositories.

If there are ethical or legal restrictions on sharing a de-identified data set, please explain them in detail (e.g., data contain potentially sensitive information, data are owned by a third-party organization, etc.) and who has imposed them (e.g., an ethics committee). Please also provide contact information for a data access committee, ethics committee, or other institutional body to which data requests may be sent. If data are owned by a third party, please indicate how others may request data access.

7. If the reviewer comments include a recommendation to cite specific previously published works, please review and evaluate these publications to determine whether they are relevant and should be cited. There is no requirement to cite these works unless the editor has indicated otherwise.

[Note: HTML markup is below. Please do not edit.]

Reviewers' comments:

Reviewer's Responses to Questions

**Comments to the Author**

1. Is the manuscript technically sound, and do the data support the conclusions?

Reviewer #1: Yes

Reviewer #2: Partly

**********

2. Has the statistical analysis been performed appropriately and rigorously?

Reviewer #1: N/A

Reviewer #2: No

**********

3. Have the authors made all data underlying the findings in their manuscript fully available?

The PLOS Data policy

Reviewer #1: Yes

Reviewer #2: No

**********

4. Is the manuscript presented in an intelligible fashion and written in standard English?

Reviewer #1: Yes

Reviewer #2: No

**********

Reviewer #1: Van Dussen et al presents the type of variants and ascertain spectrum of the Dutch Fabry disease cohort from 1999 until now, and how the spectrum of pathogenic variants unsurprisingly have shifted from mainly the ones associated with classical Fabry disease to mainly the ones associated with non-classical disease, foremost cardiomyopathy. It is interesting to know the de novo variant frequency from such a large and well-characterized cohort (8 in 319), and that many variants have likely founders (and a lot of unascertained individuals).

The article illustrates the challenge when moving from a monogenic cause of a disease (classical Fabry variants) to an oligofactorial/multifactorial disease spectrum where GLA variants is probably just a contributing part to the story, and the size of this contribution is largely unknown (but it could be major). I like their recommendation to base patient follow-up (and counselling) also on the genotype.

Major objections:

1. It is not the broader sequencing techniques that is the cause of the above-mentioned shift, but changed variant ascertainment. In theory, Sanger sequencing could have found the milder variants just as well as NGS, but since the GLA gene is included in gene panels (like for HCM), other groups of patients will get GLA variants discovered. The authors know this, but emphasis “broader sequencing techniques” as the explanation. I think it is the inclusion of GLA in gene panels that is the main explanation, i.e. GLA variant testing on weaker indications than before.

Minor comments:

1. “Neuronopathic pain” is rarely used, I prefer “neuropathic pain”.

2. Sanger sequencing was not more expensive or took longer time than today’s NGS-based testing (GLA is a small gene that can be fully sequencing after a small number of amplifications).

3. “GLA insufficiency” is mentioned in the discussion, but the reference to the article recommending the use of this term (instead of non-classical Fabry disease) is not included (even though the last author was a co-author on this recommendation).

4. The article can be shortened without loss of new information content.

Reviewer #2: Revision of the manuscript: “The changing landscape of Fabry disease; impact of the use of broad sequencing techniques on GLA variant detection and the associated phenotypes”

This manuscript presents an observational, longitudinal retrospective analysis of the Dutch national Fabry disease (FD) cohort, aiming to evaluate how the introduction of broader sequencing techniques has influenced genotype detection and phenotypic distribution. The topic is relevant and timely, particularly given the increasing use of next-generation sequencing (NGS) in clinical genetics. The dataset is valuable, and the longitudinal nature of the cohort (since 1999) represents a strength.

However, the manuscript requires substantial revision before it can be considered for publication. Major concerns relate to language quality, methodological clarity, data interpretation, and presentation of results (tables and figures). Additionally, some conclusions are speculative and insufficiently supported by data.

Major Comments

1.Language and Editorial Quality

The manuscript contains numerous grammatical, syntactical, and orthographic errors that significantly impair readability. Examples include inconsistent terminology (e.g., “alfa-galactosidase” vs “alpha-galactosidase”), incorrect punctuation (notably the use of “;” in the title), and spelling mistakes such as “arrhythmias” and “a priori.” A comprehensive English language revision by a professional editor or native speaker is strongly recommended. This should include standardization of terminology and correction of sentence structure throughout the manuscript.

2.Definition of Diagnostic Criteria

The diagnostic criteria for FD, particularly the lyso-Gb3 cut-off values, are unclear and inconsistently presented. The authors should:

• Clearly justify each cut-off value

• Reference established guidelines or prior studies supporting these thresholds

• Explain how these values were applied consistently across the cohort

3.Genetic Testing Methodology

The description of “broad sequencing techniques” lacks technical detail. Critical information is missing, including:

• Type of panels used (gene content)

• Coverage metrics (depth, sensitivity)

• Bioinformatics pipeline and variant filtering criteria

4.Results and Data Presentation

Tables and figures are incomplete and inadequately described. Specifically:

• Tables lack proper titles and explanatory legends

• Figures (e.g., Figures 1–3) are presented without sufficient captions or interpretation

• Axes, units, and statistical annotations are not clearly explained

For example, the trend shown in Figure 1 (page 20) demonstrates an increase in non-classical phenotypes over time, but no quantitative analysis of this increase is provided in the text.

All tables and figures must be:

• Fully labeled

• Referenced appropriately in the text

• Accompanied by clear legends explaining the data

5. Extrapolation of Undiagnosed Cases

The extrapolation of the number of undiagnosed individuals based on genealogical modeling (e.g., estimation from the I319T variant pedigree) is highly speculative and insufficiently validated.

While the concept of a founder effect is relevant, the current analysis lacks:

• Robust statistical modeling

• Sensitivity analyses

• Comparison with established population-based studies

The authors should either:

• Substantially strengthen this section with rigorous methodology, or

• Reduce speculative claims and present it as a hypothesis-generating observation

The work by Azevedo et al. (2020) on founder effects is the example of that research

Conclusion

This manuscript addresses an important and evolving aspect of Fabry disease diagnostics, highlighting the impact of broad sequencing techniques on phenotypic classification. However, major revisions are required to improve language quality, methodological transparency, data presentation, and scientific rigor.

In its current form, the manuscript does not yet meet the standards required for publication, but with substantial revision, it has the potential to contribute meaningfully to the field.

**********

what does this mean?). If published, this will include your full peer review and any attached files.

If you choose “no”, your identity will remain anonymous but your review may still be made public.

**Do you want your identity to be public for this peer review?**  For information about this choice, including consent withdrawal, please see our Privacy Policy

Reviewer #1: No

Reviewer #2: No

**********

[NOTE: If reviewer comments were submitted as an attachment file, they will be attached to this email and accessible via the submission site. Please log into your account, locate the manuscript record, and check for the action link "View Attachments". If this link does not appear, there are no attachment files.]

To ensure your figures meet our technical requirements, please review our figure guidelines: https://journals.plos.org/plosone/s/figures

You may also use PLOS’s free figure tool, NAAS, to help you prepare publication quality figures: https://journals.plos.org/plosone/s/figures#loc-tools-for-figure-preparation.

NAAS will assess whether your figures meet our technical requirements by comparing each figure against our figure specifications.

# Author comment: Author response to Decision Letter 1

**DOI:** 10.1371/journal.pone.0358572.r003

29 May 2026

We thank the reviewers for their valuable feedback and hope to have addressed the issues they raised satisfactory. We have addressed their comment in our document "Response to reviewers", which was uploaded separately.

Attachment Submitted filename: Rebuttal 20260528.pdf

# Peer review: Decision Letter 1

**DOI:** 10.1371/journal.pone.0358572.r004

**Authors:** Maria de Fátima Macedo

3 Sep 2026

The changing landscape of Fabry disease: impact of the inclusion of the GLA-gene in broader NGS or WES based panels on the phenotypic spectrum

PONE-D-26-06343R1

Dear Dr. Laura van Dussen,

We’re pleased to inform you that your manuscript has been judged scientifically suitable for publication and will be formally accepted for publication once it meets all outstanding technical requirements.

Within one week, you’ll receive an e-mail detailing the required amendments. When these have been addressed, you’ll receive a formal acceptance letter and your manuscript will be scheduled for publication.

An invoice will be generated when your article is formally accepted. Please note, if your institution has a publishing partnership with PLOS and your article meets the relevant criteria, all or part of your publication costs will be covered. Please make sure your user information is up-to-date by logging into Editorial Manager at Editorial Manager® and clicking the ‘Update My Information' link at the top of the page. For questions related to billing, please contact billing support.

If your institution or institutions have a press office, please notify them about your upcoming paper to help maximize its impact. If they’ll be preparing press materials, please inform our press team as soon as possible -- no later than 48 hours after receiving the formal acceptance. Your manuscript will remain under strict press embargo until 2 pm Eastern Time on the date of publication. For more information, please contact onepress@plos.org.

Kind regards,

Maria de Fátima Matos Almeida Henriques de Macedo, Ph.D.

Academic Editor

PLOS One

Additional Editor Comments (optional):

Reviewers' comments:

Reviewer's Responses to Questions

**Comments to the Author**

Reviewer #1: All comments have been addressed

**********

2. Is the manuscript technically sound, and do the data support the conclusions?

Reviewer #1: Yes

**********

3. Has the statistical analysis been performed appropriately and rigorously?

Reviewer #1: N/A

**********

4. Have the authors made all data underlying the findings in their manuscript fully available?

The PLOS Data policy

Reviewer #1: Yes

**********

5. Is the manuscript presented in an intelligible fashion and written in standard English?

Reviewer #1: Yes

**********

Reviewer #1: (No Response)

**********

what does this mean?). If published, this will include your full peer review and any attached files.

If you choose “no”, your identity will remain anonymous but your review may still be made public.

**Do you want your identity to be public for this peer review?**  For information about this choice, including consent withdrawal, please see our Privacy Policy

Reviewer #1: No

**********

# Editor report: Acceptance letter

**DOI:** 10.1371/journal.pone.0358572.r005

**Authors:** Maria de Fátima Macedo

PONE-D-26-06343R1

PLOS One

Dear Dr. van Dussen,

I'm pleased to inform you that your manuscript has been deemed suitable for publication in PLOS One. Congratulations! Your manuscript is now being handed over to our production team.

At this stage, our production department will prepare your paper for publication. This includes ensuring the following:

* All references, tables, and figures are properly cited

* All relevant supporting information is included in the manuscript submission,

* There are no issues that prevent the paper from being properly typeset

You will receive further instructions from the production team, including instructions on how to review your proof when it is ready. Please keep in mind that we are working through a large volume of accepted articles, so please give us a few days to review your paper and let you know the next and final steps.

Lastly, if your institution or institutions have a press office, please let them know about your upcoming paper now to help maximize its impact. If they'll be preparing press materials, please inform our press team within the next 48 hours. Your manuscript will remain under strict press embargo until 2 pm Eastern Time on the date of publication. For more information, please contact onepress@plos.org.

You will receive an invoice from PLOS for your publication fee after your manuscript has reached the completed accept phase. If you receive an email requesting payment before acceptance or for any other service, this may be a phishing scheme. Learn how to identify phishing emails and protect your accounts at https://explore.plos.org/phishing.

If we can help with anything else, please email us at customercare@plos.org.

Thank you for submitting your work to PLOS One and supporting open access.

Kind regards,

PLOS One Editorial Office Staff

on behalf of

Prof. Maria de Fátima Matos Almeida Henriques de Macedo

Academic Editor

PLOS One
